# Evolutionary dynamics of protein domain architecture in plants

**DOI:** 10.1186/1471-2148-12-6

**Published:** 2012-01-17

**Authors:** Xue-Cheng Zhang, Zheng Wang, Xinyan Zhang, Mi Ha Le, Jianguo Sun, Dong Xu, Jianlin Cheng, Gary Stacey

**Affiliations:** 1 Division of Plant Sciences, University of Missouri, Columbia, MO 65211 USA; 2 Department of Computer Sciences, University of Missouri, Columbia, MO 65211 USA; 3 Department of Statistics, University of Missouri, Columbia, MO 65211 USA; 4 Informatics Institute, University of Missouri, Columbia, MO 65211 USA; 5 Center for Sustainable Energy, National Center for Soybean Biotechnology, Division of Biochemistry, University of Missouri, Columbia, MO 65211 USA; 6 Department of Molecular Biology, Massachusetts General Hospital; Department of Genetics, Harvard Medical School, Boston, MA 02114 USA; 7 School of Public Health, Harvard University, Boston, MA 02115

**Keywords:** domain architecture, evolutionary dynamics, plant lineage, genetic origin

## Abstract

**Background:**

Protein domains are the structural, functional and evolutionary units of the protein. Protein domain architectures are the linear arrangements of domain(s) in individual proteins. Although the evolutionary history of protein domain architecture has been extensively studied in microorganisms, the evolutionary dynamics of domain architecture in the plant kingdom remains largely undefined. To address this question, we analyzed the lineage-based protein domain architecture content in 14 completed green plant genomes.

**Results:**

Our analyses show that all 14 plant genomes maintain similar distributions of species-specific, single-domain, and multi-domain architectures. Approximately 65% of plant domain architectures are universally present in all plant lineages, while the remaining architectures are lineage-specific. Clear examples are seen of both the loss and gain of specific protein architectures in higher plants. There has been a dynamic, lineage-wise expansion of domain architectures during plant evolution. The data suggest that this expansion can be largely explained by changes in nuclear ploidy resulting from rounds of whole genome duplications. Indeed, there has been a decrease in the number of unique domain architectures when the genomes were normalized into a presumed ancestral genome that has not undergone whole genome duplications.

**Conclusions:**

Our data show the conservation of universal domain architectures in all available plant genomes, indicating the presence of an evolutionarily conserved, core set of protein components. However, the occurrence of lineage-specific domain architectures indicates that domain architecture diversity has been maintained beyond these core components in plant genomes. Although several features of genome-wide domain architecture content are conserved in plants, the data clearly demonstrate lineage-wise, progressive changes and expansions of individual protein domain architectures, reinforcing the notion that plant genomes have undergone dynamic evolution.

## Background

Protein domains, usually segments of continuous amino acids within a protein, are the structural, functional and evolutionary units of the protein. Most proteins are composed of one or more domains that can fold independently into a stable core structure [1-3]. Each domain is usually associated with a distinct biochemical function. Protein domain architecture is a pattern of linear, sequential domain(s) in a given protein. It is often represented in diagrammatic drawings in case-by-case studies and in computer-recognizable formats in high-throughput studies. The "copy numbers" of individual domain architectures range from one to several hundred in a given genome. Usually, proteins with the same or similar architectures are close homologs, while different proteins possess distinct domain architectures.

Simple domain architectures are composed of a single domain per protein and most have been *de novo *created [4]. Complex architectures, or multi-domain architectures, have been invented by rearrangement, duplication, insertion, deletion, fusion and fission of domains [4-8]. Fong et al. (2007) reported that in multi-domain architectures, domain fusion and domain fission are the most parsimonious domain recombination events with domain fusion events occurring much more frequently compared to domain fission events [4].

Protein domain content, the overall collection of domains in a given proteome, was successfully used to reconstruct a phylogenetic tree of life that was equivalent to standard phylogenies derived from molecular sequences and phylogenomic trees based on gene content and gene order [9]. Presumably, domain architecture content, the overall collection of domain architectures in a given proteome, has a great potential to reconstruct phylogenetic trees and can be used as a probe to understand gene family expansion and the dynamics of genome evolution.

Numerous studies have sought to globally reconstruct the evolutionary history of protein domains and domain architectures by including hundreds of completed genomes [4,6,10,11]. However, the species recruited in these studies have primarily been bacteria. The significance of these studies was often limited by the relatively low level of domain architecture diversity and high rate of horizontal gene transfer among bacteria. Moreover, these studies globally grouped species into three superkingdoms, thus neglecting subtle evolutionary changes that may have occurred in more closely related lineages or taxa. By necessity, these studies were unable to correlate evolutionary behaviors of domain architectures with changes in biological processes, environmental niches, and life styles.

Numerous studies examined domain architecture evolution in the plant kingdom [12-16]. However, each of these studies focused on a specific domain architecture or a coherent group of domain architectures centered on one versatile domain and, therefore, does not provide a generalized view of protein domain architecture evolution in plants. There remains a clear need to investigate the development of protein architectures lineage by lineage over the entire history of plant evolution. We report in this study an investigation of protein domain architecture in the context of the various plant lineages, with a specific focus on the dynamics of domain architecture content and the effect of whole genome duplications.

## Results

### Genomewide distribution of domain architectures in plants

In order to construct protein domain architectures, we ran a genome-wide Pfam domain prediction of each individual protein utilizing the completed genomes of nine land plants, as well as five completed algal genomes (see Methods). The percentages of Pfam-predictable proteins in these 14 algal and plant genomes range from 42% to 75% with median 62%. Pfam-predicted architectures were further categorized into single-domain, double-domain, triple-domain and greater-than-four-domain architectures. The predominant category is single-domain architectures and the proportions of single-domain architectures per genome range from 30% (in *Physcomitrella patens*) to 51% (in *Arabidopsis thaliana*) depending on species (Table 1). Double-domain architectures constitute approximately 11% (ranging from 8% to 14%) of these genomes and triple-domain architectures 3% to 6%. Architectures containing four or more domains constitute 3% to 6% of each genome (Table 1). Beside these Pfam-predicted proteins, those proteins excluded from this analysis likely either lack Pfam annotated domains or are incorrect genome annotations. Among the Pfam-predicted architectures, the proportions of species-specific architectures detected in each genome range from 5% to 15% with median 9% (Table 1). Stated another way, each of these 14 genomes harbors approximately 9% of architectures that do not exist in any other species. This suggests that plant genomes retain a great deal of domain architecture diversity with which to generate new protein domain combinations.

**Table 1:** The profile of Pfam-predicted protein domain architectures in green plants

Domain architectures	Cr^a^	Ol	Ot	Cv	Vc	Pp	Sm	Os	Zm	Sb	Vv	At	Pt	Gm
Overall predicted^b^	0.49	0.64	0.59	0.59	0.45	0.42	0.67	0.6	0.47	0.64	0.66	0.75	0.66	0.66
Unique percentage^c^	0.09	0.05	0.06	0.09	0.12	0.07	0.09	0.15	0.15	0.09	0.12	0.06	0.12	0.10
Single-domain	0.36	0.45	0.41	0.42	0.32	0.30	0.46	0.38	0.35	0.45	0.45	0.51	0.48	0.47
Double-domain	0.09	0.13	0.12	0.11	0.09	0.08	0.12	0.11	0.08	0.11	0.12	0.14	0.12	0.12
Triple-domain	0.03	0.05	0.04	0.04	0.03	0.03	0.05	0.06	0.03	0.04	0.05	0.05	0.04	0.04
>= 4-domain^d^	0.03	0.03	0.03	0.03	0.03	0.03	0.06	0.06	0.02	0.05	0.06	0.06	0.04	0.04

^a^Species abbreviations are: Cr, *Chlamydomonas reinhardtii*; Ol, *Ostreococcus lucimarinus*; Ot, *O. tauri*; Cv, *Chlorella vulgaris*; Vc, *Volvox carteri*; Pp, *Physcomitrella patens*; Sm, *Selaginella moellendorffii*; Os, *Oryza sativa*; Zm, *Zea mays*; Sb, *Sorghum bicolor*; Vv, *Vitis vinifera*; At, *Arabidopsis thaliana*; Pt, *Populus trichocarpa*; Gm, *Glycine max*.

^b^The percentage of proteins with at least one Pfam domain predicted at an E-value cutoff of 10^-2^.

^c^Denotes the percentages of domain architectures that are unique to each species.

^d^The architectures with 4 or more domains.

Note that overall predicted architectures are categorized into single-domain, double-domain, tripe-domain and > = 4-domain architectures. The proportions of overall predicted architectures in each genome should be the sum of the proportions of these above mentioned four categories.

**Figure 1:**
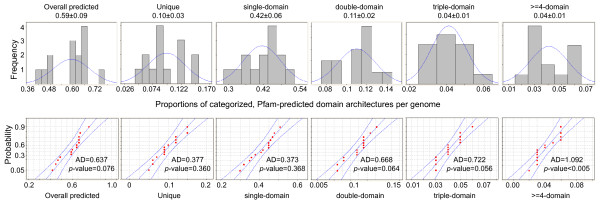
**Plant genomes maintain homogeneous distributions of protein domain architectures**. The categories of protein domain architectures, labeled on top of each histogram and on bottom of each probability plot, are overall predicted (the left panel), species-unique (second to the left panel), as well as single-domain, double-domain, triple-domain and equal to or greater than four-domain architectures (the right four panels). The numbers are mean ± standard error. The x-axis for both the upper and lower panels is the proportions of Pfam-predicted domain architectures per genome in each category. The upper panel shows frequency distributions of the percentages of these categories of domain architectures. The lower panel is the probability plot (5% significance level) of the percentages of these various categories of domain architectures across plant species. The y-axis is the probability distributions relative to the mean values. AD represents the value of Anderson-Darling normality test. Note that the proportions of domain architectures of equal to or greater than four domains do not follow a normal distribution as evidenced by the associated *p*-value in the probability plot.

Intriguingly, the proportions of overall Pfam-predicted architectures per genome are similar from species to species. Indeed, a fitted histogram shows that the proportions of overall Pfam-predicted architectures follow normal distribution (Figure 1, top panel). In parallel, the probability plot shows that these proportions follow a normal distribution. The normal distribution of these proportions is evidenced by the associated *p*-value of the Anderson-Darling normality test (0.076, greater than significant level α = 0.05) (Figure 1, lower panel). We also observed similar normal distributions of the proportions of species-unique architectures, as well as proportions of single-domain, double-domain, and triple-domain architectures (Figure 1). However, the probability of Anderson-Darling normality test of the domain architectures equal to or greater than four domains is less than 0.005 (Figure 1). Nevertheless, this conservation of probability distributions across genomes suggests that the extant green plant species roughly maintained similar genome-wide domain architecture content, even though the evolutionary history of these species spans more than 400 million years.

### Distribution patterns of domain architectures in plant lineages

Since species-to-species comparison does not necessarily reveal the general trends of domain architecture changes, we grouped the 14 green plants species into lineages and analyzed lineage-wise, dynamic changes of domain architecture content in plants. The algal lineage includes *Chlamydomonas reinhardtii *(green algae), *Ostreococcus lucimarinus*, *O. tauri*, *Chlorella vulgaris*, which are unicellular flagellates, and *Volvox carteri*, which is a multi-cellular algae. The green algae are generally considered the sister to ancestors from which land plants evolved. *Physcomitrella patens *(moss) is a bryophyte and non-vascular plant. It diverged from the lineages leading to higher plants approximately 443-490 million years ago [17]. *Selaginella moellendorffii *(spikemoss) is a lycophyte and was placed between the bryophytes and the euphyllophytes, which include ferns, gymnosperms and flowering plants. Given that both *P. patens *and *S. moellendorffii *are among the early diverging land plants and have many characteristics in common and that no other bryophyte and lycophyte genome sequences available, we grouped *P. patens *and *S. moellendorffii *together into a virtual lineage and termed this group the "early diverging lineage" in this study. The early diverging lineage is followed by the monocot lineage, which includes rice, maize and sorghum, and then the dicot lineage including grape, Arabidopsis, cottonwood, and soybean.

**Figure 2:**
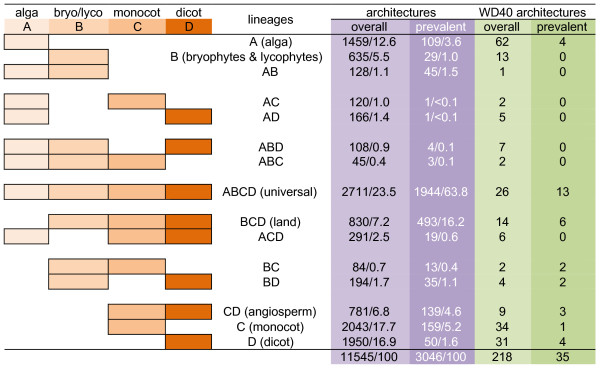
**Evolutionary dynamics of domain architectures reflected by the presence and absence of architectures in plant lineages**. Differentially colored boxes represent the presence of architecture in individual lineages or lineage combinations. Domain architecture patterns are defined by lineages or lineage combinations. Pattern A represents algal architectures; B, bryophyte and lycophyte architectures or early diverging architectures; ABCD, universal architectures; BCD, land architectures; CD angiosperm architectures; C, monocot architectures; and D, dicot architectures. Overall denotes the raw architectures without exclusion of less commonly represented architectures in each lineage and prevalent denotes architectures present in the majority of species in each lineage, i.e., at least three out of five algal species, both *P. patens *and *S. moellendorffii *species, two out of three monocot species, and three out of four dicot species. Architectures containing WD-40 domain are included as a representative to illustrate the dynamic changes in plant lineages. Numbers before the slash are collective counts of architectures in individual categories. Numbers after the slash denotes the percentages of architectures in individual categories.

We identified a total of 11545 distinct protein architectures collectively from these 14 genomes (Additional file 1, Table S1) and sorted them into 15 possible categories (Figure 2; Additional file 2, Table S2). Some categories or lineages contain thousands of distinct architectures, whereas others include only a small number of architectures (Figure 2). We also present WD40-containing architectures as an example to understand the evolution of domain architectures throughout these lineages (Figure 2; Additional file 3, Table S3).

Among the 11545 architectures, 5467 architectures are species-specific (Additional file 1, Table S1). Of the remaining 6078 architectures, most can be identified in the majority of species in each lineage. However, a large number of architectures, although found in all species in some lineages, were identified only in a single species in other lineages. For example, the architecture [SNF2_N(1) Helicase_C(1) HAND(1) SLIDE(1) ], although present in the algal, early diverging, and monocot lineages, is present only in soybean, but not in the other three dicot species. We considered this architecture less commonly represented in dicot lineages. More examples are given in Additional file 4, Table S4. To generalize the evolutionary trends and dynamics of protein domain architectures in plants and avoid the biases introduced from less commonly represented architectures in each lineage, we decided to focus only on architectures present in the majority of the species in each lineage, e.g. prevalent architectures present in at least three out of five algal species, both *P. patens *and *S. moellendorffii *species, two out of three monocot species, and three out four dicot species. This selection removed 3032 less commonly represented architectures from our analysis, while retaining 3046 prevalent architectures in our final analyses (Additional file 4, Table S4).

Among these prevalent architectures, 1944 are universally present in all four lineages, which accounts for 63.8% of the prevalent architectures (Figure 2; Additional file 4, Table S4). The conservation of these universal architectures suggests that the extant algal and land plant genomes have maintained a core set of protein components during their evolutionary history spanning a few hundred million years. For each of the four lineages, we observed a group of lineage-specific domain architectures (Figure 2; Additional file 4, Table S4). Collectively, the number of lineage-specific architectures is 347, which accounts for 11.3% of the prevalent architectures. We also observed architectures in several combinations of lineages. For example, there are 45 architectures shared in algal and early diverging lineages, 493 architectures in land plants including lineages of early diverging, monocot and dicot (land architectures), and 139 architectures in angiosperm plants including lineages of monocot and dicot (angiosperm architectures). Together with fact that ~10% of the domain architecture diversity is species-specific (Table 1), this further demonstrates that domain architecture diversity has been maintained beyond the core components of domain architectures.

### Dynamic loss and acquisition of domain architectures in the plant kingdom

The presence of species- and lineage-specific architectures suggests that these architectures were either selectively disposed of in some lineages or *de novo *created in other lineages during the course of plant genome evolution. Indeed, we observed both of these modes of protein architecture evolution (Figure 2). Apparently, 109 (3.6%) of the alga-specific architectures were disposed of in early diverging plant species. Comparably, 29 architectures specific to early diverging plants and 45 architectures present only in both the algal and the diverging lineages were lost upon the emergence of angiosperms. Due to the lack of complete plant genome sequences for comparison, firm conclusions cannot be reached concerning the fate of specific protein architectures in fern and gymnosperm plants, which are bridging lineages between early diverging and angiosperm plants. Land plants acquired 493 (14.4%) domain architectures and angiosperm plants further developed 139 (4.1%) domain architectures. Monocot and dicot plants possess 159 (4.7%) and 50 (1.5%) lineage-specific protein architectures, respectively (Figure 2; Additional file 4, Table S4).

We also observed that specific protein architectures occurred at a very low frequency in algal+early diverging+ monocot lineages (three architectures, 0.1%), algal+early diverging+dicot lineages (four architectures, 0.1%), algal+monocot lineages (one architecture, <0.1%), and algal+dicot lineages (one architecture, <0.1%) (Figure 2; Additional file 4, Table S4). It is possible that the presence of these architectures is attributable to methodological side-effects. Alternatively, these architectures might exist in extant plants but are very rare. Taking WD40-containing architectures as an example, we observed no prevalent WD40 architecture and a low incidence in the set of overall architectures in these four categories (Figure 2; Additional file 3, Table S3).

Nevertheless, the rare occurrence of domain architectures shared only in algal+early diverging+monocot lineages and algal+early diverging+dicot lineages indicates that plant genomes favored a continuous adjustment of domain architecture content. The rare occurrence of architectures shared only between algal+monocot lineages and between algal+dicot lineages indicates that plant genomes favored step-wise adjustment of domain architecture content. Interestingly, protein architectures in early diverging+monocot lineages (13 architectures, 0.4%), and early diverging+dicot lineages (35 architectures, 1.1%) occurred at a higher frequency (Figure 2; Additional file 4, Table S4), suggesting the presence of specific architectures that have undergone distinct evolutionary paths in the monocot and dicot lineages, respectively. This also holds true for the WD40-containing architectures (Figure 2; Additional file 3, Table S3). Taken together, the dynamic loss and acquisition of domain architectures suggest that plant genomes have undergone dynamic, progressive evolution.

### Genetic origins of protein domain architectures in plants

Unfortunately, at present, it is computationally challenging to perform a thorough sequence-based homology search against other kingdoms for all plant proteins. Therefore, we chose to focus on those lost and acquired architectures, i.e., protein architectures that are specific to early diverging and angiosperm plants, respectively. We noticed that most domain architectures specific to early diverging plants were also predominantly found in bacterial, fungal and primitive marine animal, and metazoan proteins, but not in plant proteins. Two prominent examples are response_reg (PF00072) and HTH_AraC (PF00165), which are involved in the bacterial two-component signal transduction system and bacterial regulation of transcription by the arabinose operon regulatory protein AraC, respectively. To investigate the origins of these proteins, we extracted the protein sequences and BLASTed these against the NCBI non-redundant protein database. It appears that a large proportion of these proteins are highly homologous, but not perfectly matched, to known bacterial, fungal and primitive marine animal proteins, which rules out the possibility of contamination of known microbial sequences during genome sequence assembly. From 640 architectures specific for the early diverging lineage, we identified a list of 243 domain architectures whose protein sequences matched to non-plant protein sequences (Additional file 5, Table S5). Among these, most were homologous to bacterial proteins (152 architectures). Most of the bacterial proteins were annotated from *Bacillus *bacteria and cyanobacteria (Additional file 5, Table S5). The next group of abundant BLASTp hits was algal proteins (28 architectures), followed by primitive marine animal proteins (16), metazoan proteins (15), fungal proteins (13) and protozoan (4). Apparently, many domain architectures are shared among early diverging plants, bacteria, fungi and primitive marine animals (Additional file 5, Table S5). Phylogenetic trees of representative domain architectures show that these early diverging proteins are indeed clustered with bacterial, fungal and primitive marine animal proteins within clades. As shown in Additional file 6, Figure S1, the top hits of a BLASTp search queried using a *P. patens *WD40 protein are bacterial and fungal sequences and this WD40 protein clusters with a couple of bacterial WD40 proteins within a clade.

To infer the origins of acquired domain architectures in angiosperm plants, we analyzed the domain architectures that are unique to lineages of angiosperm, monocot and dicot. From a total of 348 domain architectures, we identified 371 distinct domain, In contrast to the fact that these domain architectures are completely absent from algal and early diverging lineages, 302 of these 371 distinct domains are present in architectures found in algal and early diverging lineages and only the remaining 69 domains are newly emerged and present only in angiosperm plants. Likely, these newly emerged domains are plant-specific. Interestingly, these newly emerged domains are predominantly present within single-domain architectures rather than in multi-domain architectures in both monocot and dicot lineages, but not the angiosperm lineage (Table 2), consistent with previous results [4]. An good example is the dicot lineage in which 12 of 15 single-domain architectures are composed of newly created domains. In contrast to the fact that single-domain architectures dominate over multi-domain architectures in each of the seven angiosperm plants (Table 1), chi-square analyses showed that multi-domain architectures are prevalent among those architectures unique to the lineages of angiosperm, monocot and dicot, respectively (Table 2). As exemplified by the WD40-containing architectures, the great majority of WD40 architectures in angiosperm, monocot and dicot lineages are composed of multiple domains (Additional file 3, Table S3). Furthermore, chi-square analyses also showed that multiple-domain architectures combining pre-existing domains are significantly overrepresented (Table 2), consistent with previous reports [4,18-20]. Collectively, these data suggest that the newly emerged domains in higher plants are predominantly present in single-domain architectures and higher plants have a tendency to integrate pre-existing domains to execute combinational functions, perhaps in response to increased cellular complexity.

**Table 2:** Angiosperm plants tend to integrate pre-existing domains into multi-domain architectures

		lineages		overall
		
	angiosperm	monocot	dicot	angiosperm^a^
Single-domain	65 (46.7%)	51 (37.2%)	15 (30%)	1124771 (69.2%)^e^
Newly emerged/existing domain^bc^	33/32	11/40	12/3	
Multiple-domain	74 (53.3%)	86 (62.8%)	35 (70%)	55402 (30.8%)^e^
Newly emerged/existing domain^d^	15/59	4/82	8/27	
Chi-square test^f^	0.015 (*p *= 0.7)	16.49 (*p *< 0.001)	5.4 (*p *< 0.05)	
Chi-square test	26.16 (*p *< 0.001)	70.74 (*p *< 0.001)	10.91 (*p *< 0.001)	
Chi-square test^h^	23.75 (*p *< 0.001)	48.04 (*p *< 0.001)	72.09 (*p *< 0.001)	

^a^Includes the 3 monocot species, Os, Zm, and Sb, and the 4 dicot species, Vv, At, Pt, and Gm.

^b^New domains are defined as domains that are not present in algal and early diverging lineages. Pre-existing are domains that are present in algal and early diverging lineages.

^c^Indicates the numbers of new and pre-existing domains in single-domain architectures in the three lineages examined.

^d^Indicates the numbers of new and pre-existing domains in multiple-domain architectures in the three lineages examined.

^e^Denotes the sum and percentage of single-domain and multiple-domain architectures collectively from all 7 angiosperm species.

^f^Chi-square values for the expected ratios of 50% newly emerged: 50% existing domains in multiple-domain architectures in single-domain architectures.

^g^Chi-square values for the expected ratios of 50% newly emerged: 50% existing domains in multiple-domain architectures.

^h^Chi-sqaure values for the expected ratio of 69.2% single-domain architectures: 30.8% multipl-domain architectures.

### Genomic dosages of protein domain architectures in plants

**Figure 3:**
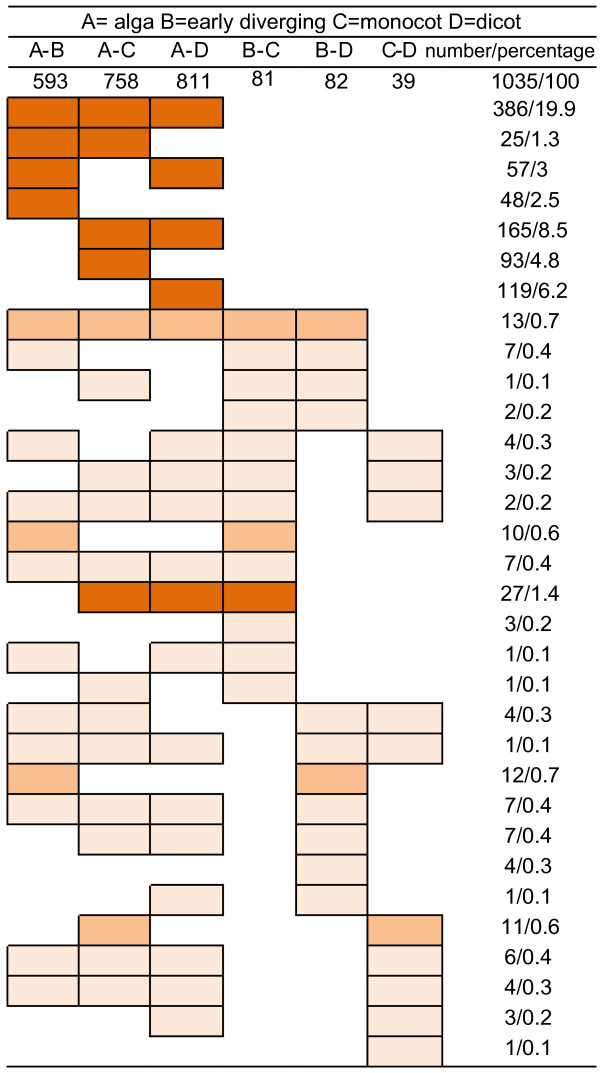
**Lineage-wise architecture expansion in plants**. Pairwise comparisons of genomic dosages of architectures were made between lineages and colored boxes represent the significant expansion of architectures. Patterns of more than 25 counts of architectures are shown in red and less than 25 in light orange. The numbers denotes the counts and percentages of architectures of each pattern that have undergone significant expansion. Only the patterns that have an incidence higher than 1% are shown.

One of the hallmark characteristics of plant evolution is successive rounds of whole genome duplication (WGD) [21-23], which inevitably leads to expansion of numerous gene families. Examples are presented in Additional file 7, Table S6. We observed an unequivocal expansion, i.e., increase of copy numbers per genome, of many protein architectures among the 1944 universal domain architectures and the 493 domain architectures unique to land plants (Figure 3; Additional file 8, Table S7). To further investigate protein architecture expansion, we performed T-tests for genomic dosages of all universal domain architectures with multiplication adjustments. Our analyses showed that a total of 1035 out of 1944 universal architectures show a clear trend of expansion at the significance level of 1% (Figure 3; Additional file 8, Table S7). For example, 386 (19.9%) universal architectures, including four WD40-containing architectures (Additional file 3, Table S3), expanded from algal species to early diverging, monocot and dicot lineages. In comparison, 165 (8.5%) universal architectures did not significantly expand from algae to early diverging plants, but their occurrence did expand in monocot and dicot plants (Figure 3; Additional file 8, Table S7). We also observed differential expansion in three groups of domain architectures, although they all expanded from algae to early diverging species. Among these architectures, 48 (2.5%) show no trend of expansion in both monocot and dicot lineages, 25 (1.3%) expanded further from early diverging species to monocot species only, and 57 (3%), including two WD40-containing architectures (Additional file 3, Table S3), expanded further from early diverging species to dicot species only. Similarly, 93 (4.8%) did not expand from algae to early diverging species, but expanded in monocot species only and 119 (6.2%) did not expand from algae to early diverging species, but show expansion in dicot species only. These data suggest that some domain architectures underwent discriminating expansion in monocot and dicot lineages, respectively. In other words, land plants specifically retained some duplicated domain architectures, while specifically discriminating against the retention of other duplicated domain architectures.

Compared to the massive domain architecture expansion from algal species to higher plants (Figure 3; Additional file 8, Table S7), the expansions from early diverging species to angiosperm plants and from monocot to dicot species are almost imperceptible. Only 23 (1.1%) protein architectures show expansion from early diverging species to both monocot and dicot species (Figure 3; Additional file 8, Table S7). Likewise, 58 (2.9%) protein architectures expanded from early diverging to monocot species only and 36 (1.8%) expanded from early diverging to dicot species only. This indicates that the domain architecture content was generally established by the expansion from algal to early diverging species with relatively minor changes during the development of higher plants.

Presumably, the expansion of protein domain architectures in higher plants can be largely explained by high rate of retention of duplicated genes after rounds of WGD. This suggests that the observed expansion of universal architectures would not be apparent if each higher genome was normalized to a presumed ancestral genome that has not undergone WGD. Most early diverging plants, such as *P. patens*, are haploid-dominant during most of their life cycle, while angiosperm plants have undergone rounds of WGD [21-23]. Assuming that most duplicated domain architectures were completely or highly retained, the genomic dosages of architectures are the exponential expression of the numbers of WGD. We normalized the universal domain architecture content in angiosperm plants by dividing the number of distinct domain architectures per genome by factors of 8 (three rounds of WGD with complete retention) and 16 (four rounds of WGD with complete retention), while leaving the architecture content in early diverging plants unmodified. As a result of this normalization, T-test analyses showed that essentially none of the universal domain architectures had undergone expansion in angiosperm plants. Indeed, the general trend is toward a slight reduction in the number of universal protein architectures as a function of the presumed ancestral genome (Additional file 8, Table S7). Similarly, a decrease in the number of protein architectures is also apparent for land-specific lineages when normalized to the presumed ancestral genome. These data suggest that the observed expansion in the number of domain architectures is largely explained by WGD and that the retention of duplicated domain architectures is incomplete. However, our analyses cannot exclude a number of other factors (e.g., genome size, selection pressure and local rearrangement) that could impact the retention of specific protein architectures.

While the majority of protein domain architectures appear to have expanded during plant evolution, we also identified 909 universal architectures whose genome content remained roughly unchanged throughout plant evolution at the significance level of 1% (Additional file 8, Table S7). However, at the significance level of 5%, 334 universal architectures were found to have undergone moderate expansion (Additional file 8, Table S7). We also identified 241 architectures whose genome content is less than or equal to two and 14 architectures that are predominantly present as single-copy domain architectures in land plants (Additional file 8, Table S7). Compared to the general trend of expansion of protein domain architectures, it is likely that negative selection after WGD maintained dosages of specific protein domain architectures at levels equivalent to the presumed ancestral genome.

## Discussion

### Lineage-based domain architecture content as a probe to study plant genome evolution

Since protein domains define specific, conserved biochemical functions, they tend to be more highly conserved than entire protein sequences. This feature makes domain architecture content, like domain content [9], an ideal probe to study protein domain evolution and genome evolution. The evolutionary history of protein domain and domain architectures has been extensively studied in microorganisms. For example, various studies described the distribution of single-domain and multi-domain architectures [4-6,18,24], domain architecture convergence and divergence [6,25], domain duplication [25,26], and domain co-occurrence [27-29]. Most of these studies focused solely on a broad analysis of the three superkingdoms, archaea, prokaryotes and eukaryotes [4,9,18]. However, studies at the superkingdom level are less informative in reconstructing detailed histories of domain architecture evolution than at successive, flexible levels such as phylum and lineage.

By introducing the concept of lineage, we show that lineage-by-lineage changes of domain architectures reflect the evolutionary dynamics of domain architectures in plants. Although many domain architectures are universally present in all plant lineages, a large proportion of architectures are lineage-specific (Figure 2; Additional file 4, Table S4). By taking advantage of both lineage-based species grouping and domain architecture content (over sequence-based similarity search), we succeeded in probing the evolutionary history of plant genomes by focusing on the lineage-based dynamics of domain architecture content. This study can inspire similar approaches to understand the evolutionary dynamics of other kingdoms or lineages, especially the metazoa whose genomes have also undergone rounds of WGD [30].

Although Pfam prediction is commonly accepted, it does not predict all structural and functional motifs and domains. To determine how extensive the Pfam coverage in predicting all domains in this study, we decided to run COILS [31] for left-handed coiled-coils and PreDisorder [32] for disordered region on those 8,509 (~25%) non-Pfam-predictable proteins in the Arabidopsis genome, a widely used reference plant genome. Our results showed that on average 0.74% of the residues in these proteins are predicted to be left-handed coiled-coil and 42.5% disordered. The average probability of disordered residues is 0.654 and the average *p*-value of coiled-coil residues is 0.0008, which are above the probability threshold of 0.5 for disordered region and lower than that of 0.01 for coiled-coils, respectively. The low existence rate of coiled-coils in non-Pfam-predictable Arabidopsis proteins suggests that coiled-coil domain families likely are well represented in the Pfam database for PfamScan execution. For example, Pfam domain PF05710 is one type of coiled-coil structural unit. On the other hand, the presence of a higher proportion of disordered residues may explain why these proteins are recalcitrant to predictions by Pfam, as well as other commonly used domain methods. Although no data are available for other plant genomes, it is reasonable that non-Pfam-predictable proteins in other examined genomes would harbor a large portion of disordered regions and a low incidence of coiled-coil units.

Most genome annotations are not experimentally validated. Even for those well-annotated genomes, like Arabidopsis, it changes from version to version. To what extent genome annotation errors affect the genome-wide domain architecture content is still an open question. To answer this question, we analyzed domain architecture content built on different versions of Arabidopsis annotation. The original analysis was based on the TAIR8 annotation and we reconstructed the domain architecture content of TAIR7 by subtracting domain architectures newly added in TAIR8 followed by adding domain architectures deleted in TAIR8. Expressed another way, TAIR7 = TAIR8-TAIR8new+TAIR8deleted. Subtraction of 222 TAIR8new architectures from TAIR8 dataset did not eliminate any architecture except for one of the 1944 universal architectures (ABCD) in re-constructed TAIR7 dataset (Additional file 9, Table S8). The changes mainly involve the copy numbers of individual architecture only by one or few. The pattern of lost and gained domain architectures in the TAIR7 dataset appears to be very similar to that in TAIR8 dataset (Additional file 9, Table S8). Similarly, this is true for comparisons between TAIR8 and TAIR6 and between TAIR8 and TAIR9 datasets. In general, we did not observe significant difference of domain architecture content between TAIR6, 7, 8 and 9 annotations. Likely, this will be the similar case for other genomes and the genome-wide domain architecture content will largely be similar between different versions of annotations of most, if not all, plant genomes.

### Distribution of single-domain and multi-domain architectures

Distribution of single-domain and multi-domain architectures is an essential part of studies on the evolutionary history of domain architecture content. Our data indicate that the proportions of single-domain proteins in algal and land plants examined range from 30% to 51% (mean ± SE, 42% ± 6%) and that of multi-domain architectures range from 13% to 25% (19% ± 1%) (Table 1). This distribution is consistent with a previous study in which single-domain architectures comprised more than 42% of all architectures [4]. However, these results are contradictory to other published studies [5,6,8,18,24]. In those studies, multi-domain architectures comprised over two-thirds of all architectures; whereas single-domain architectures only constituted less than one-third of all architectures. It is likely that these differences are due to the incorporation of a much higher number of bacterial species in these latter studies. Because the majority of bacterial proteins, such as found in *Mycoplasma genitalium *[8], contain more than one domain, sampling of genomes enriched in bacterial species would likely yield a skewed distribution of single- and multi-domain architectures.

### Conserved features and domain architecture diversity of plant genomes

Several aspects of lineage-based domain architecture content are evolutionary conserved in plants. Despite the fact that proteome sizes vary among species, the percentages of Pfam-predictable proteins in each plant proteome are very close (Table 1). This also holds true for the percentages of species-unique architectures, single-domain architectures and multi-domain architectures (Table 1). In addition, the majority (~65%) of the prevalent domain architectures are universally present in all plant lineages. Finally, the genomic abundance of approximately one-half of the universal domain architectures is constrained at a steady level and did not expand significantly. For example, there are 14 universal architectures that are single-copy in all land plants (Additional file 8, Table S7).

Lineage-based domain architecture content has undergone dynamic changes in plants. The first line of evidence comes from the presence of species- and lineage-specific domain architectures. Species-specific domain architectures range from 5% to 15% (mean ± SE, 10% ± 3%) (Table 1). Moreover, the lineage-specific architectures collectively account for 11.5% of prevalent domain architectures in plants (Figure 2; Additional file 4, Table S4). The presence of species-specific architectures indicates both the loss and *de novo *creation of domain architectures in higher plants. Consistent with gene expansion following rounds of WGD [21-23], over 50% of the universal architectures showed a lineage-specific expansion (Figure 3; Additional file 8, Table S7). Although this expansion was largely established in early diverging plants (likely the first ancestor of land plants), an unequivocal trend of expansion was observed in angiosperm plants (Figure 3; Additional file 8, Table S7). However, a decrease of domain architecture content is apparent in angiosperm genomes when the number of architectures is normalized to a presumed ancestral genome (Additional file 8, Table S7), suggesting that domain architecture expansion was largely due to the WGD and retention of duplicated domain architectures was incomplete.

### Genetic origins of domain architectures in plants

Since domain architecture is a more stringent parameter than protein sequence similarity in classifying homologous proteins, we utilized the domain architecture content to examine the origins of plant proteins. Our data indicate that a collection of 243 domain architectures unique to early diverging plants are also present in bacteria, alga, fungi, primitive marine animals, protozoa, and metazoa (Additional file 5, Table S5). Although more direct evidence is needed, sequence and phylogenetic analyses of representative domain architectures demonstrate horizontal gene transfer (HGT) and genetic flows into the plant kingdom. There are probably at least two major genetic flows. Hypothetically, the first reflects genetic flow from cyanobacteria into the latest common ancestor (LCA) of land plants via both alga-dependent and alga-independent mechanisms (Additional file 5, Table S5). The second reflects the genetic flow from bacteria into the LCA of land plants, consistent with the endosymbiosis theory [33-36]. Likely, a group of architectures flowed from bacteria into both the LCA of land plants and the LCA of primitive marine animals in parallel, which eventually reached into the LCA of metazoa. It is commonly accepted that the bacteria involved in endosymbiosis process are mainly proteobacteria and cyanobacteria [33-36]. Intriguingly, sequences derived from *Bacillus *bacteria substantially predominated over those from proteobacteria and cyanobacteria in this study; a result for which we have no plausible explanation but may simply reflect limited sampling size (Additional file 5, Table S5).

By analyzing the domain architectures unique to angiosperm plants, we show that newly emerged domains are primarily present in single-domain architectures rather than in multi-domain architectures (Table 2). According to Fong et al., these new domains likely are *de novo *created and not the breakdown products of multi-domain architectures. Interestingly, multi-domain architectures tend to combine pre-existing domains, rather than to create novel domains, a point that is consistent with previous studies [4,11,18,20]. Generally, there are no more than two newly created domains in multi-domain architectures. This indicates that the complex events required to combine multiple new domains simultaneously are rare.

## Conclusions

Our data show that single-domain proteins account for one third to a half of the entire proteome; whereas multi-domain proteins account for one sixth to one fifth of the entire proteome. Analyses of the lineage distribution of domain architectures show that approximately 65% of domain architectures are universally present in all 14 green plant genomes. The conservation of these universal architectures suggests that the extant algal and land plant genomes have maintained a core set of protein components during their evolutionary history spanning a few hundred million years. In contrast, the presence of lineage-specific protein domain architectures, especially those lost or gained in higher plants, demonstrates that domain architecture diversity has been maintained beyond the core components of domain architectures. As expected, the acquired multi-domain architectures mainly arose from recombinations of pre-existing domains. In general, there has been a dynamic, lineage-wise expansion of domain architectures in plant lineages. However, the data suggest that this expansion can be largely explained by whole genome duplications. By introducing the concept of lineage, we show that lineage-by-lineage changes of domain architectures reflect the evolutionary dynamics of domain architectures in plants. This strategy could be exemplary to our understanding of evolutionary dynamics of protein domain architectures in the animal kingdom.

## Methods

### Protein sequence collection

The protein sequences were retrieved from the following databases: green algae (*Chlamydomonas reinhardtii*; http://genome.jgi-psf.org/Chlre3/Chlre3.home.html); *Ostreococcus lucimarinus *(http://genome.jgi-psf.org/Ost9901_3/Ost9901_3.home.html); *O. tauri *(http://genome.jgi-psf.org/Ostta4/Ostta4.home.html); *Chlorella vulgaris *(http://genome.jgi-psf.org/Chlvu1/Chlvu1.home.html); *Volvox carteri *(http://genome.jgi-psf.org/Volca1/Volca1.home.html); moss (*Physcomitrella patens*; http://genome.jgi-psf.org/Phypa1_1/Phypa1_1.home.html); spikemoss (*Selaginella moellendorffii*; http://genome.jgi-psf.org/Phypa1_1/Phypa1_1.home.html); rice (*Oryza sativa*; http://rice.plantbiology.msu.edu/osa1.shtml#); maize (*Zea mays*; http://magi.plantgenomics.iastate.edu/downloadall.html); sorghum (*Sorghum bicolor*; http://genome.jgi-psf.org/Sorbi1/Sorbi1.home.html); grape (*Vitis vinifera*; http://www.genoscope.cns.fr/externe/GenomeBrowser/Vitis/); Arabidopsis (*Arabidopsis thaliana*; http://www.arabidopsis.org/); poplar (*Populus trichocarpa*; http://genome.jgi-psf.org/Poptr1_1/Poptr1_1.home.html); soybean (*Glycine max*; http://www.phytozome.net/soybean).

### Domain prediction and construction of domain architectures

In order to predict domains, we ran Pfam prediction locally [37]. We also locally installed NCBI BLAST [38], HMMER [39], and PfamScan program [37]. PfamScan predicts proteins by systematically executing BLAST and HMMER to search against domain profiles stored in the Pfam databases and produced an e-value for each of the predicted domains. We used 10^-2 ^as a cutoff value. The PfamScan output files of the 14 species were then parsed by a computer program to construct domain architectures. For example, the architecture of a protein containing three LysM motifs and one protein-kinase domain, in the order of from N-terminus to C-terminus of the protein, was recorded as "LysM(3) PKinase (1)". All domain architecture analyses were automatically performed by a pipeline using PERL programming language. The final domain architecture dataset was input into MS Office Excel and lineage-based domain architecture content was manually sorted based on the lineages and the copy numbers of domain architectures.

### Protein sequence search and sequence analysis

The protein sequences corresponding to domain architectures in early diverging lineage were extracted and used as query sequences to BLAST against the NCBI non-redundant protein database at an E-value cutoff of E-10. The protein sequences of candidate proteins were extracted and aligned using MUSCLE3.6 [40] with default settings and a FASTA output format and manually edited using Jalview [41]. Majority-ruled parsimonious trees were generated using the program "protpars" of PHYLIP [42] using the following parameters: random number seed = 3, times of jumble = 3, data set = 1000.

### Statistical analysis

The histograms and probability plots of the proportions of Pfam-predictable and species-unique domain architectures were performed using MINITAB 15. The fitness of distributions was judged by the smallest value Anderson-Darling goodness-of-fit statistic and the associated *p*-value higher than the chosen α-level of 0.05. Pearson's chi-square tests were performed following standard procedures. For lineage-based domain architecture expansion, we normalized the architecture content of maize genome and soybean genome by dividing with factors of 3 and 2, respectively, because the annotation of maize genome was not masked and the soybean genome underwent a nascent WGD around 13 million years ago [43]. Lineage-specific expansion of domain architecture content was analyzed by T-tests. For each domain architecture, T-tests were performed on the logarithm-transformed genomic dosages of architectures. The domain architecture content was analyzed simultaneously and the *p*-values were adjusted by BOOTSTRAP.

## Authors' contributions

XCZ, DX, JC, and GS conceived this study. XCZ, ZW, and MHL collected data, carried out analyses. JS provided statistical advice. XZ carried out statistical analyses. XCZ wrote the draft manuscript. DX, JC, and GS discussed the results and contributed to manuscript revisions. All authors read and approved the final manuscript.

## Supplementary Material

Additional file 1 **Overall and species-specific domain architectures in green plants**. This file contains all Pfam-predicted domain architectures. The "overall" tab lists 11545 domain architectures predicted from all 14 plant genomes included in this study. The remaining tabs list the species-specific domain architectures. Click here for file

Additional file 2 **Distributions of overall domain architectures in plant lineages**. Pfam-predicted domain architectures (listed in Additional file 1, Table S1) were sorted into different plant lineages or lineage combinations. Pattern A represents algal architectures; B, bryophyte and lycophyte architectures or early diverging architectures; ABCD, universal architectures; BCD, land architectures; CD angiosperm architectures; C, monocot architectures; and D, dicot architectures. Click here for file

Additional file 3 **Distribution of WD40-containing domain architectures in plant lineages**. Domain architectures containing WD40 domain are represented as an example in plant lineages or lineage combinations. Click here for file

Additional file 4 **Distribution of prevalent domain architectures in plant lineages**. This file lists all architectures present in the majority of the species in each lineage, e.g. prevalent architectures present in at least three out of five algal species, both *P. patens *and *S. moellendorffii *species, two out of three monocot species, and three out four dicot species. Click here for file

Additional file 5 **Genetic origins of early diverging domain architectures**. BLASTp searches using protein sequences of early diverging-specific domain architectures show the presence of highly homologous sequences in non-plant species, including bacteria, fungi, ancient marine animals, and metazoan. This suggests a possible common ancestor of these sequences before splitting of bacteria, fungi, plants and animals. Click here for file

Additional file 6 **Genetic origin of a *P. patens *WD 40 protein**. A WD40 architecture in *P. patens *is homologous to bacterial sequences as supported by a BLASTP search against NCBI database using the P. patens WD40 protein as query (top hits are all bacterial and fungal sequences ) and by a majority- ruled parsimony tree with maximum- likelihood branch length 9 the *P. patens *WD40 protein cluster together with bacterial sequences). Click here for file

Additional file 7 **Example domain architectures illustrating architecture expansion in green plants**. Expansion of domain architectures in plants illustrated by representative architectures, including Myb_DNA-binding (2), F-box(1), as well as TIR (1). The right six columns indicate the pairwise comparison between lineages of the probability of domain architecture expansion during the plant genome evolution. Click here for file

Additional file 8 **Expansion of universal and land domain architectures**. Expansion of universal and land domain architectures (BCD) was shown in individual tabs. Domain architectures that have not undergone expansion are also shown. Click here for file

Additional file 9 **Categorical distribution of domain architectures in TAIR8 and presumed TAIR6, 7, and 9 annotations**. The effect of genome annotation errors on the genome-wide domain architecture content was examined by analyzing genome-wide domain architecture content built on different version of Arabidopsis annotations, which are thought to be well annotated. In general, we did not observe significant difference of domain architecture content between TAIR6, 7, 8 and 9 annotations. Click here for file
