# Supplementary material for: Evolutionary dynamics of protein domain architecture in plants
Source: BMC Evol Biol. 2012 Jan 17;12:6. doi: 10.1186/1471-2148-12-6 (PMC3310802; doi:10.1186/1471-2148-12-6)
Supplement: Additional file 6 — Genetic origin of a P. patens WD 40 protein. A WD40 architecture in P. patens is homologous to bacterial sequences as supported by a BLASTP search against NCBI database using the P. patens WD40 protein as query (top hits are all bacterial and fungal sequences ) and by a majority- ruled parsimony tree with maximum- likelihood branch length 9 the P. patens WD40 protein cluster together with bacterial sequences). [file 1471-2148-12-6-S6.PDF]

Sequences producing significant alignments:

Accession	Description	Max score	Total score	Query coverage	E value	Links
XP_001773244.1	predicted protein [Physcomitrella patens subsp. patens] >gb	1351	1351	100%	0.0	G
YP_001865069.1	hypothetical protein Npun_F1419 [Nostoc punctiforme PCC 7	139	762	44%	2e-31	G
ZP_08426695.1	WD-40 repeat-containing protein [Lyngbya majuscula 3L] >(	133	552	43%	2e-29	
YP_004447017.1	(myosin heavy-chain) kinase [Haliscomenobacter hydrossis	130	1035	43%	2e-28	G
YP_322074.1	ribosome assembly protein 4 [Anabaena variabilis ATCC 294	130	943	44%	2e-28	G
XP_002483884.1	WD-repeat protein, putative [Talaromyces stipitatus ATCC 1	127	818	44%	2e-27	G
XP_002340131.1	G-protein beta WD-40 repeats containing protein, putative [	126	919	46%	3e-27	G
EGO54028.1	hypothetical protein NEUTE1DRAFT_124378 [Neurospora tet	125	441	40%	3e-27	
YP_004451364.1	WD40 repeat-containing protein [Haliscomenobacter hydros	123	349	40%	1e-26	G
YP_003300732.1	metallophosphoesterase [Thermomonospora curvata DSM 4	122	900	40%	4e-26	G
ZP_06973979.1	WD40 repeat, subgroup [Ktedonobacter racemifer DSM 449(	115	210	42%	6e-26	
ZP_06972304.1	serine/threonine protein kinase with WD40 repeats [Ktedonc	120	326	43%	9e-26	
YP_001661272.1	serine/threonine protein kinase [Microcystis aeruginosa NIES	120	223	41%	1e-25	G
XP_001940843.1	vegetative incompatibility protein HET-E-1 [Pyrenophora trit	120	474	41%	2e-25	G
XP_001221064.1	hypothetical protein CHGG_01843 [Chaetomium globosum C	119	466	45%	5e-25	G
ZP_08492181.1	WD40 repeat-containing protein [Microcoleus vaginatus FGP-	118	543	47%	7e-25	
ZP_01620255.1	WD-40 repeat protein [Lyngbya sp. PCC 8106] >gb|EAW378	119	659	44%	7e-25	
XP_001588570.1	hypothetical protein SS1G_10117 [Sclerotinia sclerotiorum 1	118	654	40%	9e-25	G

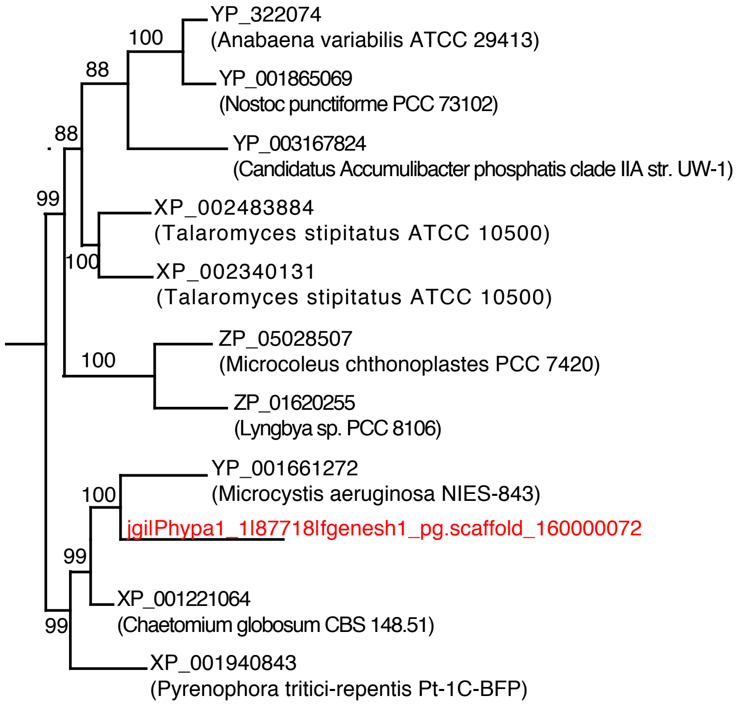

Figure S1. A WD40 architecture in *P. patens* is homologous to bacterial sequences. Top panel: screenshot of an BLASTp search against NCBI using the *P. patens* WD40 as query. Note that top hits are all bacterial and fungal sequences. Left panel shows a majority-ruled parsimony tree with maximum-likelihood branch length. Note that the *P. patens* WD40 protein cluster together with bacterial sequences.
