# Supplementary material for: Evolutionary dynamics of protein domain architecture in plants
Source: BMC Evol Biol. 2012 Jan 17;12:6. doi: 10.1186/1471-2148-12-6 (PMC3310802; doi:10.1186/1471-2148-12-6)
Supplement: Additional file 7 — Example domain architectures illustrating architecture expansion in green plants. Expansion of domain architectures in plants illustrated by representative architectures, including Myb_DNA-binding (2), F-box(1), as well as TIR (1). The right six columns indicate the pairwise comparison between lineages of the probability of domain architecture expansion during the plant genome evolution. [file 1471-2148-12-6-S7.PDF]

Table S5. Example domain architectures illustrating architecture expansion in green plants.

	A= alga					B=bryo & lyco		C=monocot			D=dicot				Probability					
architectures	Cr[a]	Ol	Ot	Cv	Vc	Pp	Sm	Os	Zm	Sb	Vv	At	Pt	Gm	A-B[d]	A-C	A-D	B-C	B-D	C-D
Myb_DNA-binding(2)[b]	10[c]	10	8	5	13	49	41	116	80	111	124	145	201	151	[e]					
Peptidase_C48(1)	7	2	2	2	5	8	10	294	82	41	3	11	12	12						
F-box(1)	12	5	6	5	15	135	180	502	98	360	76	164	188	171						
Pkinase(1)	368	75	74	126	267	384	650	827	573	495	583	551	868	695						
WRKY(2)	0	0	0	0	0	3	6	17	12	9	10	17	24	17						
Auxin_inducible(1)	0	0	0	0	0	18	33	57	17	68	74	77	99	137						
TIR(1)	0	0	0	0	0	8	1	1	2	1	26	37	115	38						
DUF677(1)	0	0	0	0	0	3	7	3	3	3	5	18	11	11						
Kunitz_legume(1)	0	0	0	0	0	0	0	1	0	1	5	7	25	25						

[a]Species abbreations are: Cr, Chlamydomonas reinhardtii; Ol, Ostreococcus lucimarinus; Ot, O. tauri; Cv, Chlorella vulgaris; Vc, Volvox carteri; Pp, Physcomitrella patens; Sm, Selaginella moellendorffii; Os, Oryza sativa; Zm, Zea mays; Sb, Sorghum bicolor; Vv, Vitis vinifera; At, Arabidopsis thaliana; Pt, Populus trichocarpa; Gm, Glycine max.

[b]Architectures are represented by domains in the order they appear from N- to C-terminus in a given protein. The numbers in the parenthesis indicate the copy numbers of the domain.

[c]Numbers are the additive copy number of each distinct protein architectures in a given genome.

[d]A-B indicates the pairwise comparison between lineages of the probability of protein architecture expansion according to non-parametric one-way ANOVA analysis.

[e]Probability of architecture expansion between lineages with a 99% confidence cutoff. Architectures undergone significant expansion is highlighted in grey.
